# Secondhand exposure to e-cigarette aerosols among smokers: A cross-sectional study in six European countries of the EUREST-PLUS ITC Europe Surveys

**DOI:** 10.18332/tid/99117

**Published:** 2019-02-15

**Authors:** Olena Tigova, Beladenta Amalia, Yolanda Castellano, Marcela Fu, Sarah O. Nogueira, Christina N. Kyriakos, Ute Mons, Antigona C. Trofor, Witold A. Zatoński, Krzysztof Przewoźniak, Tibor Demjén, Yannis Tountas, Anne C. K. Quah, Geoffrey T. Fong, Esteve Fernández, Constantine I. Vardavas, on behalf of the EUREST-PLUS consortium

**Affiliations:** 1 Catalan Institute of Oncology (ICO), L’Hospitalet de Llobregat, Spain; 2 Bellvitge Biomedical Research Institute (IDIBELL), L’Hospitalet de Llobregat, Spain; 3 School of Medicine and Health Sciences, University of Barcelona, Barcelona, Spain; 4 European Network for Smoking and Tobacco Prevention (ENSP), Brussels, Belgium; 5 University of Crete (UoC), Heraklion, Greece; 6 Cancer Prevention Unit and WHO Collaborating Centre for Tobacco Control, German Cancer Research Center (DKFZ), Heidelberg, Germany; 7 University of Medicine and Pharmacy ‘Grigore T. Popa’ Iasi, Iasi, Romania; 8 Aer Pur Romania, Bucharest, Romania; 9 Health Promotion Foundation (HPF), Warsaw, Poland; 10 European Observatory of Health Inequalities, President Stanisław Wojciechowski State University of Applied Sciences, Kalisz, Poland; 11 Maria Skłodowska-Curie Institute-Oncology Center (MSCI), Warsaw, Poland; 12 Smoking or Health Hungarian Foundation (SHHF), Budapest, Hungary; 13 National and Kapodistrian University of Athens (UoA), Athens, Greece; 14 Department of Psychology and School of Public Health and Health Systems, University of Waterloo (UW), Waterloo, Canada; 15 Ontario Institute for Cancer Research, Toronto, Canada

**Keywords:** e-cigarette, secondhand exposure, secondhand aerosol, passive exposure, Europe

## Abstract

**INTRODUCTION:**

Electronic cigarette (e-cigarette) use has grown significantly in some European Union (EU) Member States (MS). A better understanding of the exposure to secondhand e-cigarette aerosols (SHA) is necessary to develop and implement comprehensive regulations on e-cigarette use in public places. This study aims to assess the observation of e-cigarette use in public places, the self-reported exposure to SHA, and the level of users’ comfort using e-cigarettes in the presence of others.

**METHODS:**

This is a cross-sectional study of the Wave 1 International Tobacco Control 6 European Countries Survey recruiting adult smokers (n=6011) across six EU MS: Germany, Greece, Hungary, Poland, Romania, and Spain, within the EURESTPLUS Project. A descriptive analysis was conducted to estimate the prevalence (%) of observed e-cigarette use in different places, frequency of self-reported exposure to SHA, and level of comfort using e-cigarettes in the presence of others.

**RESULTS:**

In all, 31.0% of smokers observed others using e-cigarette in public places, 19.7% in indoor places where smoking is banned, and 14.5% indoors at work. Almost 37% of smokers reported to be ever exposed to SHA, ranging from 17.7% in Spain to 63.3% in Greece. The higher prevalence of observed e-cigarette use and passive exposure to SHA was reported by smokers of younger age, of higher educational level and those being current or former e-cigarette users. Part (8.8%) of the smokers who were also e-cigarette users reported feeling uncomfortable using e-cigarettes in the presence of others.

**CONCLUSIONS:**

A third of smokers from six EU MS reported being exposed to SHA. Prevalence differences were observed among the countries. In the context of scarce evidence on long-term health effects of exposure to SHA, precautionary regulations protecting bystanders from involuntary exposure should be developed.

## INTRODUCTION

Electronic cigarettes (e-cigarettes) are relatively new in the market of nicotine and tobacco products; nevertheless, awareness about these devices and prevalence of their use is growing in some European Union (EU) Member States (MS)^1^. While the health effects of active e-cigarette use have received substantial attention in research, evidence concerning the impact on the health of bystanders exposed to secondhand e-cigarette aerosols (SHA) is relatively scarce^2^.

SHA (mainstream aerosol exhaled by e-cigarette users) contains a number of toxic components, among them: propylene glycol, glycerol, formaldehyde, acetaldehyde, nicotine, particulate matter, polyaromatic hydrocarbons, volatile organic compounds (VOC), tobacco-specific nitrosamines, acrolein, and metals such as copper, cadmium, nickel and lead^3^. The World Health Organisation (WHO) suggests that even though it is unknown whether exposure to the toxic components of SHA leads to an increased risk of morbidity and mortality among bystanders, the epidemiological evidence of the negative health effects of some of its components (e.g. fine particles and nicotine) is well-established and cannot be neglected^4^.

A population-based study conducted in Italy in 2017 found that 13.7% of e-cigarette non-users were exposed to SHA in various indoor settings on a daily basis^5^. This shows that despite the relatively low prevalence of active e-cigarette use (1.1%) in that country, passive exposure was considerably high^5^. This might be that in many public places the use of e-cigarettes is still not regulated and these devices are used in settings where the use of combustible tobacco cigarettes is forbidden^6^.

The Seventh Session of the Conference of Parties to the WHO Framework Convention on Tobacco Control (FCTC) has suggested some policy options for countries to regulate e-cigarette use in public places; one is to ban the use of e-cigarettes in areas where smoking is prohibited^7^. As of October 2017, 20 EU MS had national regulations regarding e-cigarette use in public places^8^, and whilst the prevalence of use is monitored^1^, there is almost no information about exposure of bystanders.

Knowledge of the actual exposure of the population to SHA is necessary to formulate comprehensive regulations on e-cigarette use in public places. Therefore, the objective of this study was to improve current knowledge on exposure to SHA in Europe with regard to observed e-cigarette use in public places, self-reported exposure to e-cigarette aerosols, and degree of users’ comfort near other people.

## METHODS

### Design

This study is part of the *European Regulatory Science on Tobacco: Policy implementation to reduce lung diseases* (EUREST-PLUS) Project that aims to evaluate the impact of the EU Tobacco Products Directive (TPD)^9^ within the context of the WHO FCTC^10^ ratification at a European level. One of the objectives of the EUREST-PLUS Project is to assess the psychosocial and behavioural impact of TPD and FCTC implementation through the inception of a longitudinal cohort of approximately 6000 smokers as part of the International Tobacco Control survey (ITC) across six EU MS (ITC 6E)^11^. The participating countries of the EUREST-PLUS ITC 6E cohort are: Germany (n=1003), Greece (n=1000), Hungary (n=1000), Poland (n=1006), Romania (n=1001), and Spain (n=1001). These countries have both diverse prevalence and regulation of e-cigarette use in public places (Table 1).

**Table 1:** National regulation on the e-cigarette use in public places in six European Union Member States

*Country*	Ever e-cigarette use (%)*	Current e-cigarette use (%)*	Legislation in place**	Year of legislation**	Comments**
Germany	8	2	No	N/A	No regulation in place
Greece	9	3	Yes	2010	The regulation for a ban on smoking in public places includes e-cigarettes. A petition by the Association of Greek E-cigarettes Businesses was rejected by Greece’s highest administrative court in decision 704/2018, upholding that Greece laws on conventional smoking also apply to e-cigarettes.
Hungary	6	1	Yes	2016	The ban concerns almost the same venues in which tobacco smoking is banned with the exception of areas designated for smoking.
Poland	9	1	Yes	2016	Use of e-cigarettes is banned in all indoor public places, including hospitality venues and workplaces. The ban concerns almost the same venues in which tobacco smoking is banned.
Romania	7	0	No	N/A	No regulation in place.
Spain	9	1	Yes	2014	Use of e-cigarettes is banned in all indoor public places, with the exception of hospitality venues. The ban includes use of e-cigarettes in outdoor areas of schools, healthcare organizations and children’s playgrounds.

* 2017 Eurobarometer Data^15^

** Institute for Global Tobacco Control^8^.

The information was reviewed and extended by local researchers of the study.

Data in this study were derived from the baseline (Wave 1) survey that was carried out from June to September 2016, recruiting current smokers (having smoked >100 cigarettes in their lifetime and having smoked at least once in the past 30 days) age ≥18 years and using a multistage stratified sample representative of all geographical regions in each EU MS. A random walk method was used to select eligible households where, if possible, male and female smokers were chosen using the Next Birthday method for interview^12^. All interviews were conducted using tablets (computer-assisted personal interviewing) after informed consents from participants were obtained. The details of the sampling and survey methods of this ITC 6E Wave 1 Survey have been presented elsewhere^13^. The study protocol received approval from an ethics committee in each of the participating countries and institution partners, and was registered at Clinicaltrials.gov (registration number NCT02773836).

### Measures

Observed e-cigarette use in public places was described based on sites where participants saw someone using e-cigarettes. The observed e-cigarette use in public places was ascertained from participants who knew about e-cigarettes by the question: ‘In the last 30 days, how often have you seen someone using an e-cigarette or vaping device in public (excluding you)?’. Participant answers: ‘Everyday’, ‘Most days’, or ‘Some days’ were recoded as ‘Yes’; while ‘Rarely’ and ‘Not at all’ were recoded as ‘No’. Those respondents who answered ‘Yes’ were subsequently asked about observed e-cigarette use ‘indoors where smoking ordinary cigarettes is banned’ with the question: ‘In the last 30 days, have you seen someone using e-cigarettes or vaping devices indoors where smoking ordinary cigarettes is banned?’. Possible answers were: ‘No’, ‘Yes, but only once’, ‘Yes, a few times’, and ‘Yes, frequently’. ‘No’ answers from this and the previous question were recorded as ‘No’ and the last three responses were recoded as ‘Yes’. All respondents who knew about e-cigarettes and worked outside home were asked about observed e-cigarette use at indoor workplaces with the question: ‘In the last 30 days, have people used an e-cigarette or vaping device in indoor areas where you work?’, with possible answers ‘Yes’ or ‘No’.

E-cigarette use status was determined with the question: ‘Have you ever used an e-cigarette or vaping device, even one time?’. The participants who responded ‘No’ were classified as ‘never users’. If they answered ‘Yes’, a subsequent question was asked: ‘On average, how often do you currently use e-cigarettes or vaping devices?’; with possible answers: ‘Daily’, ‘Less than daily, but at least once a week’, ‘Less than weekly, but at least once a month’, ‘Less than monthly’ (all previous answers recoded as ‘current user’), and ‘Not at all’ (recoded as ‘former user’).

Self-reported frequency of exposure to SHA was ascertained by asking non-current e-cigarette users (former and never users) with the question: ‘How often are you exposed to the vapour from other people’s e-cigarettes or vaping devices?’; with possible answers: ‘Never’, ‘Rarely’, ‘Sometimes’, ‘Often’, and ‘Very often’.

Comfortability with e-cigarette use around other people was determined by asking participants who used e-cigarettes at least once a month: ‘How comfortable do you feel about using e-cigarettes or vaping devices around other people?’; with possible answers ‘Very comfortable’, ‘Comfortable’, ‘Neutral’, ‘Uncomfortable’, and ‘Very uncomfortable’. Receiving negative reactions from others to their e-cigarette use was also assessed among the same participants by asking: ‘In the last 30 days, have you received any negative reactions to your using e-cigarettes or vaping devices from any of the following groups: ‘Strangers’, ‘Work colleagues’, ‘Friends’, and ‘Family’?’. Response options were ‘Yes’ or ‘No’ for each group.

Sociodemographic characteristics studied were country, sex (male, female), age group (18–24, 25– 39, 40–54, ≥55 years old), and level of education (low: primary, lower pre-vocational secondary, middle pre-vocational secondary; moderate: secondary vocational, senior general secondary and pre-university; and high: higher professional and university Bachelor, university Masters).

### Analysis

A descriptive analysis was conducted to estimate the prevalence (%) and 95% confidence intervals (CI) of observed e-cigarette use in different places (in public in general, in indoor places where smoking conventional cigarettes is banned, and in indoor areas of workplaces) and frequency (% and 95% CI) of SHA exposure according to sociodemographic characteristics (country, sex, age groups, and level of education) and e-cigarette use status. Additionally, comfortability with e-cigarette use around other people and receiving negative reactions to e-cigarette use were assessed using Pearson’s chi-squared test. Statistical significance was set at p<0.05. All analyses applied the sample weights to account for the complex sample design and were conducted using STATA version 13.0.

## RESULTS

### Observed e-cigarette use

Among all smokers from six EU MS who had ever knew about e-cigarettes, 31.0% had seen people using e-cigarettes in public in the last 30 days, and 19.7% observed people using e-cigarettes in indoor places where smoking conventional cigarettes was banned (Table 2). The highest level of observed e-cigarette use was reported in Greece: 55.2% in public and 40.5% in indoor areas where smoking was prohibited. The lowest prevalence of observed e-cigarette use was reported in Spain and Hungary: 12.3% and 16.3% in public, 5.7% and 9.2% in indoor areas where smoking was banned, respectively.

**Table 2:** Prevalence of observed e-cigarette use in different settings across six European Union Member States, 2016

	*In public (n=4142)*					*Indoors where smoking is banned (n=4122)*					*Indoor areas at work (n=2566)*				

	*N*	*n*	*%*	*95% CI*	p*	*N*	*n*	*%*	*95% CI*	p*	*N*	*n*	*%*	*95% CI*	p*
**All**	4142	1244	31.0	(28.5 - 33.5)		4122	804	19.7	(17.8 - 21.6)		2566	362	14.5	(12.6 - 16.4)	
**Country**					<0.001					<0.001					<0.001
Germany	629	174	28.2	(22.9 - 33.5)		628	95	15.4	(11.5 - 19.3)		428	35	9.6	(5.7 - 13.6)	
Greece	734	416	55.2	(45.9 - 64.5)		733	330	40.5	(33.7 - 47.4)		440	172	37.4	(30.3 - 44.5)	
Hungary	652	95	16.3	(9.8 - 22.7)		651	50	9.2	(4.3 - 14.1)		457	19	5.7	(1.3 - 10.0)	
Poland	646	283	45.4	(39.8 - 51.1)		631	190	31.4	(26.1 - 36.7)		381	64	15.3	(11.9 - 18.8)	
Romania	651	170	29.2	(23.8 - 34.6)		650	88	15.8	(11.6 - 20.1)		386	55	16.2	(11.6 - 20.7)	
Spain	830	106	12.3	(9.0 - 15.6)		829	51	5.7	(3.7 - 7.6)		474	17	4.0	(1.8 - 6.1)	
**Sex**					<0.001					0.009					0.007
Male	2178	706	33.6	(30.7 - 36.5)		2173	457	21.3	(19.0 - 23.5)		1500	235	15.5	(13.3 - 17.8)	
Female	1964	538	27.5	(24.8 - 30.2)		1949	347	17.5	(15.3 - 19.8)		1066	127	12.8	(10.3 - 15.3)	
**Age (years)**					<0.001					<0.001					0.004
18-24	379	127	34.7	(28.2 - 41.1)		377	91	24.5	(18.3 - 30.8)		207	35	19.8	(11.0 - 28.6)	
25-39	1269	432	33.8	(30.4 - 37.2)		1263	285	21.9	(19.0 - 24.8)		942	146	15.3	(12.6 - 18.0)	
40-54	1405	419	29.8	(26.8 - 32.8)		1396	275	19.0	(16.5 - 21.5)		1026	148	14.7	(12.2 - 17.2)	
≥55	1089	266	27.1	(22.9 - 31.2)		1086	153	15.0	(12.1 - 18.0)		391	33	7.5	(5.1 - 9.9)	
**Level of education**					<0.001					<0.001					<0.001
Low	1472	353	25.3	(21.6 - 29.0)		1470	201	14.0	(11.2 - 16.8)		800	81	11.4	(8.2 - 14.7)	
Moderate	2149	683	32.6	(29.7 - 35.6)		2135	456	21.3	(18.9 - 23.7)		1380	204	14.9	(12.6 - 17.2)	
High	498	197	40.5	(35.5 - 45.6)		494	141	29.6	(24.6 - 34.6)		370	74	20.2	(16.0 - 24.3)	
**E-cigarette use status**					<0.001					<0.001					<0.001
Current	174	119	67.2	(59.5 - 74.9)		173	74	41.0	(33.4 - 48.5)		106	29	27.3	(18.6 - 36.0)	
Former	989	386	38.0	(34.5 - 41.5)		985	262	26.2	(22.8 - 29.6)		630	130	22.8	(18.6 - 26.9)	
Never	2973	737	26.3	(23.4 - 29.2)		2958	466	16.0	(14.0 - 18.0)		1827	202	10.8	(8.9 - 12.7)	

CI: Confidence Intervals.

* p-value: Pearson’s chi-squared test

Among all respondents who knew about e-cigarettes and worked outside home (Table 2), 14.5% had observed in the last 30 days someone using e-cigarettes in indoor areas where they work. Again, the highest prevalence was reported in Greece (37.4%) and the lowest in Spain (4.0%) and Hungary (5.7%).

Observed e-cigarette use was reported more frequently by male respondents, by younger age participants and those with higher level of education (Table 2). Noticing someone using e-cigarette in public was more frequently reported by current e-cigarette users (67.2%). Differences in prevalence among all groups analysed (by country, age, sex, level of education and e-cigarette use status) were all statistically significant (Table 2).

### Self-reported frequency of exposure to SHA

Among all respondents in six EU MS, 177 reported currently using e-cigarettes and 1000 were former users. Most smokers from six EU MS who did not report currently using e-cigarettes declared being never exposed to SHA produced by others (63.3%). Nevertheless, some of them were exposed to SHA rarely or sometimes (33.1%) and 3.6% declared to be exposed often or very often (Table 3). The respondents from Greece and Poland were the ones who reported being exposed to SHA often or very often the most, 7.3% and 5.6%, respectively; while in Spain this prevalence was the lowest (1.2%).

**Table 3:** Self-reported frequency of secondhand e-cigarette aerosol exposure among smokers (non-current e-cigarette users) in six European Union Member States (N=3979), 2016

	*N*	*Never*				*Rarely or sometimes*				*Often or very often*			

		*n*	*%*	*95% CI*	*p*	*n*	*%*	*95% CI*	*p*	*n*	*%*	*95% CI*	*p*
**All**	3979	2550	63.3	(60.7 - 65.8)		1279	33.1	(30.7 - 35.5)		150	3.6	(3.0 - 4.3)	
**Country**					<0.001				<0.001				<0.001
Germany	579	411	69.6	(63.7 - 75.5)		157	28.7	(23.1 - 34.4)		11	1.7	(0.5 - 2.8)	
Greece	696	250	36.7	(28.2 - 45.4)		387	56.0	(47.7 - 64.3)		59	7.3	(4.9 - 9.6)	
Hungary	642	499	79.2	(74.2 - 84.1)		129	18.7	(14.4 - 23.0)		14	2.1	(0.9 - 3.3)	
Poland	615	274	43.9	(37.8 - 50.0)		303	50.5	(44.5 - 56.5)		38	5.6	(3.8 - 7.4)	
Romania	630	454	66.6	(60.8 - 72.5)		160	29.6	(24.1 - 35.1)		16	3.8	(1.9 - 5.7)	
Spain	817	662	82.3	(77.4 - 87.3)		143	16.5	(11.6 - 21.3)		12	1.2	(0.4 - 2.0)	
**Sex**					0.008				0.030				0.179
Male	2094	1302	61.1	(58.1 - 64.1)		705	34.8	(32.0 - 37.7)		87	4.1	(3.2 - 5.0)	
Female	1885	1248	66.1	(63.2 - 69.0)		574	30.9	(28.2 - 33.6)		63	3.0	(2.2 - 3.8)	
**Age (years)**					<0.001				<0.001				0.026
18-24	344	205	60.2	(54.2 - 66.3)		121	35.2	(29.4 - 40.9)		18	4.6	(2.2 - 6.9)	
25-39	1225	724	59.2	(55.4 - 63.0)		453	36.9	(33.4 - 40.5)		48	3.9	(2.6 - 5.2)	
40-54	1349	851	64.1	(60.9 - 67.3)		439	31.9	(29.0 - 34.8)		59	4.0	(2.8 - 5.1)	
≥55	1061	770	69.3	(64.9 - 73.7)		266	28.5	(24.1 - 32.9)		25	2.2	(1.3 - 3.2)	
**Level of education**					<0.001				<0.001				<0.001
Low	1427	1045	73.6	(70.4 - 76.8)		349	24.5	(21.3 - 27.8)		33	1.9	(1.2 - 2.6)	
Moderate	2053	1238	58.6	(55.5 - 61.7)		715	36.5	(33.6 - 39.4)		100	4.9	(3.9 - 6.0)	
High	476	253	51.7	(46.2 - 57.2)		207	44.9	(39.7 - 50.1)		16	3.4	(1.5 - 5.2)	
**E-cigarette use status**					<0.001				<0.001				<0.001
Former	982	557	55.5	(51.9 - 59.1)		369	38.4	(34.9 - 41.8)		57	6.1	(4.7 - 7.6)	
Never	2991	1993	66.0	(63.1 - 68.8)		906	31.2	(28.6 - 34.0)		92	2.8	(2.1 - 3.4)	

CI: Confidence Intervals. *p-value: Pearson’s chi-squared test

Slightly higher prevalence of rare or sometimes exposure to SHA was declared among males than females (34.8% vs 30.9%, respectively) (Table 3). Overall, any SHA exposure was more frequently reported among younger age groups (18–54 years) and by respondents with moderate and higher level of education. With regard to previous experience of e-cigarette use, the respondents who were former users declared more frequently to be exposed to SHA (44.5%) compared to never users (34.0%; Table 3).

### Comfort level of using e-cigarettes around other people

Among participant smokers who also used e-cigarettes (dual users) at least once per month (n=109), 43.1% reported feeling comfortable or very comfortable using e-cigarette around other people, 48.1% felt neutral and 8.8% uncomfortable or very uncomfortable (Table 4). The highest prevalence of the participants who declared feeling comfortable or very comfortable when they used e-cigarettes around other people was observed in Greece (59.1%) and Romania (48.7%), and the lowest in Hungary (26.1%) and Poland (28.6%). In all countries, except Greece and Romania, most users felt neutral when using e-cigarettes in the presence of others.

**Table 4:** Comfort level of using an e-cigarette around other people (n=109), 2016

	*N*	*Comfortable or very comfortable*			*Neutral*			*Uncomfortable or very uncomfortable*		

		*n*	*%*	*95% CI*	*n*	*%*	*95% CI*	*n*	*%*	*95% CI*
**All**	109	49	43.1	(32.4 - 53.8)	51	48.1	(37.6 - 58.6)	9	8.8	(3.0 - 14.6)
**Country**										
Germany	31	13	39.4	(22.3 - 56.6)	17	58.1	(40.2 - 75.9)	1	2.5	(0.0 - 6.9)
Greece	31	21	59.1	(37.3 - 81.0)	7	26.5	(6.5 - 46.3)	3	14.4	(0.0 - 30.8)
Hungary	12	3	26.1	(0.0 - 52.3)	9	73.9	(47.7 - 100)	0	0	-
Poland	13	3	28.6	(5.6 - 51.5)	7	57.1	(31.9 - 82.4)	3	14.3	(0.0 - 29.5)
Romania	15	6	48.7	(12.6 - 84.7)	7	36.4	(4.7 - 68.2)	2	14.9	(0.0 - 33.0)
Spain	7	3	31.7	(0.0 - 72.2)	4	68.3	(27.8 - 100)	0	0	-
**Negative reactions to e-cigarette use from:**										
Strangers	7	2	24.5	(0.0 - 54.4)	3	39.0	(3.7 - 74.2)	2	36.5	(2.2 - 70.9)
Work colleagues	5	2	54.9	(14.1 - 95.7)	2	31.2	(0.0 - 67.7)	1	13.9	(0.0 - 38.6)
Friends	13	7	60.7	(36.8 - 84.7)	4	26.5	(6.5 - 46.4)	2	12.8	(0.0 - 28.4)
Family	10	6	56.7	(26.0 - 87.5)	2	30.2	(0.0 - 61.5)	2	13.1	(0.0 - 28.5)

CI: Confidence Intervals

Overall, 24 users of e-cigarettes reported receiving in total 35 negative reactions from different groups of bystanders about their e-cigarette use. Among them, 15 respondents reported receiving negative reaction from one bystander group, seven users received negative reactions from two different bystanders’ groups, and two users from three groups. In total, most of the negative reactions came from friends and family (Table 4).

## DISCUSSION

Overall, about a third of the respondents in our sample of cigarette smokers from six EU MS observed other people using e-cigarettes in public; about 20% observed e-cigarette use in indoor places where smoking conventional cigarettes was forbidden. Approximately 15% of respondents observed people using e-cigarettes at indoor workplaces. These results, overall as well as the country differences, are in line with self-reported frequency of exposure to SHA among those not using e-cigarettes, with slightly more than a third of respondents declaring to be exposed to the aerosols from e-cigarettes. These observations are also consistent with the differences in overall e-cigarette use prevalence among the countries included in current research^1,14,15^ and general support for the ban of e-cigarette use in public places^16^.

The findings of the current study reflect a similar range of the potential level of exposure to SHA in public places as those reported for the UK and Australia, where 34% and 13%, respectively, of the respondents declared using e-cigarettes in smoke-free public places^17^. Moreover, the observed e-cigarette use in indoor workplaces ranged from 4.0% in Spain to 37.4% in Greece, suggesting significant differences between the countries studied and potentially high levels of exposure to e-cigarette SHA at workplaces in some countries. These figures are in broad agreement with the parallel prevalence of exposure to secondhand tobacco smoke (SHS) in these countries^18^. An internet survey conducted in Japan that analysed e-cigarette use in restaurants and workplaces reported that almost 30% of the respondents used e-cigarettes in smoke-free restaurants and about a quarter in smoke-free workplaces^19^. In five out of six countries in the current study the observed e-cigarette use is lower than that observed in Japan. Nevertheless, exposure at these venues still exists, creating potentially harmful environments for bystanders. This also denotes a failure to comply with national regulations in the countries where such are in place. Taking into account that the TPD does not harmonise the rules on aerosol-free settings^9^, national regulations and their compliance play an important role in creating such environments.

Existing evidence indicates that exposure to SHA is not harmless^20^ and a significant proportion of bystanders may be exposed to toxicants from aerosols. One study using biomarkers of passive exposure reported that non-smokers passively exposed to SHA absorb nicotine^21^, and tobacco specific nitrosamines have been detected in the body fluids of bystanders exposed to SHA^22^. Another study estimated that, while the computed disability-adjusted life years attributed to SHA were relatively lower compared to those attributed to SHS and thirdhand smoke exposures, these were comparable with SHS and thirdhand smoke exposure for some components such as VOCs^23^. Therefore, regulations of e-cigarette use in public places should be developed and introduced to protect bystanders from involuntary secondhand and thirdhand exposure to aerosols^24-26^. Moreover, comprehensive aerosol-free policies are of importance to strengthen existing smoke-free laws as the use of e-cigarettes in public places may renormalise tobacco smoking, maintain dual use, and ultimately weaken previous efforts to create smoke-free environments^24,27^.

Taking into account that in four out of six countries included in the study legislation prohibiting e-cigarette use in public places is currently in force (Table 1); strengthening existing legislations and adherence to them should be stressed. Greece, where regulation on e-cigarette use came into force in 2010 (Law 3868/2010)^28^, is the country with the highest prevalence of observed e-cigarette use in public places and reported SHA exposure. This may reveal other hidden aspects hindering adherence to current legislation, such as low level of enforcement and penalties application, degree of local government involvement, and tobacco industry investment in novel tobacco products^29,30^.

Similar to the findings on observed e-cigarette use, exposure to SHA was also reported more frequently among younger age groups and among smokers with higher educational level. These determinants are similar to those observed for e-cigarette use and awareness about e-cigarettes^14^. Children and youth are considered one of the most vulnerable groups to SHS^31,32^. Regarding socioeconomic determinants of SHS exposure, a number of studies have shown that higher exposure is associated with lower level of education^31,33^. These differences could be attributed to the distinct sociodemographic characteristics of e-cigarette users. A few studies from the United States and Europe observed higher odds of ever e-cigarette use among adults with higher educational level^34,35^. This may suggest that bystanders exposed to SHA and active users of e-cigarettes share similar sociodemographic characteristics, different from those of active and passive smokers. This observation is in agreement with diffusion of innovation theory, according to which innovators and early adopters of new behaviours are male, with higher socioeconomic status compared to later adopters^36^, as previously observed for the spread of smoking^37^. Further research on these determinants is warranted to tailor policy regulations protecting bystanders from exposure to SHA that may be different from the policies targeting SHS.

The majority of the smokers who also reported to be currently using e-cigarettes declared feeling neutral about e-cigarette use around other people. This is the case for all countries except Greece and Romania, where the majority of the users felt comfortable or very comfortable using e-cigarettes in the presence of bystanders, reaching 60% in Greece. This, on one hand, may reflect low levels of awareness about the health impact of SHA exposure among users and a lack of enforcement of current regulations^38-40^. On the other hand, this situation may represent a window of opportunity to increase awareness about potential harms among e-cigarette users and the general public^41^. Previous research indicates that perceived harm of exposure to SHA on individual health is associated with support of e-cigarette use restrictions in public spaces^42^; therefore, ultimately the efforts on increasing knowledge about the health effects of SHA exposure may increase support for aerosol-free policies and improve overall adherence to them^43^.

That many e-cigarette users felt comfortable or neutral to use e-cigarettes in the presence of bystanders may also indicate that e-cigarette use around others has been deemed as socially acceptable by the users. The variability of comfort level towards using e-cigarettes around other people at the country level could be attributed to the degree of peer influence on e-cigarette experimentation and use, and also the different regulatory environment of e-cigarette use and adherence to it that may promote more favourable attitudes towards e-cigarette use and its greater occurrence in public settings^17,44,45^.

When interpreting the descriptive results about feeling comfortable with using e-cigarettes around other people and receiving negative reactions from others, one should take into account that the overall absolute numbers in these categories are very small. Nevertheless, the existing evidence in these aspects is scarce and should not be neglected. These results may indicate that those respondents who felt comfortable using e-cigarettes in the presence of others, overall receive more negative reactions than those feeling neutral or uncomfortable. This might be explained by the fact that the respondents feeling comfortable using e-cigarettes in the presence of others do it more often and, therefore, are more likely to receive more complaints. Also, these respondents received more complaints from family and friends rather than strangers. This may be explained by the settings where e-cigarettes were more commonly used and also by higher social acceptability towards e-cigarette use in some countries. However, interpretation of these findings should be made with caution due to the small number of respondents and also the cross-sectional nature of the survey, although reverse association is unlikely in this case.

The current study has further limitations. The sample comprised current cigarette smokers and, therefore, the results cannot be generalised to the general populations of the six countries in scope, but to the adult smoker population. Also, exposure to SHA was gathered only from non-users of e-cigarettes (former or never users) omitting the current e-cigarette users; therefore, exposure to SHA among smokers may be underestimated as the current e-cigarette users could be also exposed to SHA and this information was not collected; nonetheless, there were only 177 users of e-cigarette reporting current use. The comfort level of e-cigarette use around others was based on e-cigarette users in our samples of smokers and cannot be generalized to all e-cigarette users (comprising also those who do not concurrently smoke conventional cigarettes). This work is based on the Wave 1 of the cohort study and has a cross-sectional nature; therefore, precluding any causal inference. However, the study is based on national representative samples of smokers in each country, providing valuable insights for this population. Given that smokers and e-cigarette users are less in favour of restrictions to e-cigarette use, it is of particular importance to gain a better understanding of exposure levels among this population and, therefore, obtain more insights for policy makers^43^. The reported results based on a sample of smokers might be a better estimation of overall SHA exposure, given that awareness about e-cigarettes is usually higher among smokers^35^ and they are more likely to notice e-cigarette use in their surroundings. Furthermore, the current study was conducted in six EU MS, providing knowledge on countries with different profiles (e-cigarette use prevalence, smoke-free and aerosol-free regulation, etc.). Even though this study is cross-sectional, this is the Wave 1 of the longitudinal study and will provide the basis for further trends analysis and prospective characterisation of SHA exposure determinants. Finally, this survey is based on a large ITC survey, enabling cross-country comparisons in the future.

## CONCLUSIONS

A third of European smokers observed e-cigarette use in public places and a fifth at indoor areas where smoking is prohibited. The use of e-cigarettes at indoor areas of workplaces was also observed. More than a third of smokers were exposed to SHA. These findings suggest that a non-negligible part of the European population is exposed to SHA. The development and enforcement of public policies creating aerosol-free areas is necessary to protect bystanders’ health.
